# Emotional face processing across neurodevelopmental disorders: a dynamic faces study in children with autism spectrum disorder, attention deficit hyperactivity disorder and obsessive-compulsive disorder

**DOI:** 10.1038/s41398-020-01063-2

**Published:** 2020-11-02

**Authors:** Marlee M. Vandewouw, EunJung Choi, Christopher Hammill, Paul Arnold, Russell Schachar, Jason P. Lerch, Evdokia Anagnostou, Margot J. Taylor

**Affiliations:** 1 Department of Diagnostic Imaging, Hospital for Sick Children, Toronto, Canada; 2 Program in Neurosciences & Mental Health, Hospital for Sick Children, Toronto, Canada; 3 Bloorview Research Institute, University of Toronto, 150 Kilgour Road, Toronto, Canada; 4 Mathison Centre for Mental Health Research & Education, Hotchkiss Brain Institute, Cumming School of Medicine, University of Calgary, Alberta, Canada; 5 Department of Psychiatry, Hospital for Sick Children, Toronto, Canada; 6 Wellcome Centre for Integrative Neuroimaging, FMRIB, Nuffield Department of Clinical Neurosciences, University of Oxford, Oxford, UK; 7 Department of Medical Biophysics, University of Toronto, Toronto, Canada; 8 Department of Psychology, University of Toronto, Toronto, Canada; 9 Department of Medical Imaging, University of Toronto, Toronto, Canada

**Keywords:** Neuroscience, Psychology

## Abstract

Autism spectrum disorder (ASD) is classically associated with poor face processing skills, yet evidence suggests that those with obsessive-compulsive disorder (OCD) and attention deficit hyperactivity disorder (ADHD) also have difficulties understanding emotions. We determined the neural underpinnings of dynamic emotional face processing across these three clinical paediatric groups, including developmental trajectories, compared with typically developing (TD) controls. We studied 279 children, 5–19 years of age but 57 were excluded due to excessive motion in fMRI, leaving 222: 87 ASD, 44 ADHD, 42 OCD and 49 TD. Groups were sex- and age-matched. Dynamic faces (happy, angry) and dynamic flowers were presented in 18 pseudo-randomized blocks while fMRI data were collected with a 3T MRI. Group-by-age interactions and group difference contrasts were analysed for the faces vs. flowers and between happy and angry faces. TD children demonstrated different activity patterns across the four contrasts; these patterns were more limited and distinct for the NDDs. Processing happy and angry faces compared to flowers yielded similar activation in occipital regions in the NDDs compared to TDs. Processing happy compared to angry faces showed an age by group interaction in the superior frontal gyrus, increasing with age for ASD and OCD, decreasing for TDs. Children with ASD, ADHD and OCD differentiated less between dynamic faces and dynamic flowers, with most of the effects seen in the occipital and temporal regions, suggesting that emotional difficulties shared in NDDs may be partly attributed to shared atypical visual information processing.

## Introduction

Face processing deficits are widely reported in psychiatric populations and negatively affect family and social relationships^1^. Although autism spectrum disorder (ASD) is classically associated with poor face processing skills^2^, increasing evidence shows emotional and social-cognitive impairments in obsessive-compulsive disorder (OCD) and attention deficit hyperactivity disorder (ADHD)^3,4^. As these neurodevelopmental disorders (NDDs) can be comorbid and share overlapping symptoms^5–7^, there are common difficulties in cognitive domains. Beyond comorbidity, NDDs can share common cognitive deficits but the underlying pathophysiology might be different. We investigated brain function underpinning face processing, contrasting fMRI measures of dynamic emotional face processing across these three clinical paediatric groups, compared with typically developing (TD) controls in a large, single-site cohort.

Facial expressions of emotion arise from facial movements and are rich sources of social information. Recognizing and understanding emotional expressions are essential for appropriate social behaviour. We typically process faces rapidly with minimal attentional resources^8,9^, being very effective at discerning emotions from facial movements. Behavioural^10^ and neuroimaging studies^11–14^ show that, compared to static expressions (i.e., photographs), dynamic facial expressions convey compelling information that is more similar to what we encounter in everyday social interactions. Dynamic presentation of facial emotions improves identification of emotion^15,16^ and increases the ecological validity^12–14,17^. Nevertheless, static photographs of facial expressions have been predominantly used in imaging studies.

In response to static facial expressions, activity is seen in core face-processing regions, including fusiform gyri, amygdalae and temporal cortices, with the fusiform and the superior temporal sulci (STS) being implicated in the detailed perception of faces^9,18–20^. These same areas are active to dynamic faces, particularly the STS^21^ and V5^11–13,22^. However, dynamic faces also include activation in frontal areas, including inferior and orbital frontal gyri^14,22^. Normative studies with dynamic faces found increases in core face-processing regions, consistent with increased salience, with frontal increases consistent with greater social-cognitive processing. Given the increased ecological validity and salience of dynamic faces, these stimuli are beginning to be used in clinical populations with emotional processing difficulties. Below we review briefly the neuroimaging literature on dynamic emotional face processing in ASD, OCD and ADHD.

## ASD

The classic work of Kanner^23^ described emotional abnormalities in autism, which have since been confirmed by many studies^24–26^. Social communication difficulties are a key symptom of ASD, and central to social interactions is understanding emotions and their expression.

Structural and functional imaging studies have found abnormalities in brain regions associated with emotional face processing in ASD (e.g.^27–29^). Studies including the use of dynamic faces in ASD are, however, almost exclusively behavioural. Enticott et al.^30^ reported that dynamic faces improved recognition of angry but not sad faces in adults with ASD, while Zane et al.^31^ reported that children with ASD did not show the same sensitivity to positive or negative valence with dynamic faces as controls. In teenagers with ASD, Law Smith et al.^32^ found reduced accuracy in identifying emotional expressions, particularly at a lower intensity, despite them being dynamic, similarly to Weiss et al.^33^ in adolescents and adults with ASD. However, one fMRI study^34^ reported that with dynamic faces there were no activation differences between adults with and without ASD. Given the wealth of other neuroimaging data showing group differences in face and emotional face processing, and the evidence that those with ASD experience difficulties with understanding emotions, we expected to find abnormalities in activation to emotional faces in this population, but that group differences may decrease with age.

## OCD

Emotional dysfunction is often considered a key component in OCD, with emphasis on recognition of disgust^35,36^. Daros et al.^37^ completed a meta-analysis and found that across ten behavioural studies, those with OCD were less accurate in recognising emotional faces, particularly disgust and anger. Others reported OCD patients had lower social-cognitive awareness^38^ and poorer performance on a facial recognition task^39^.

Few neuroimaging studies have explored emotional face processing in OCD, showing either enhanced face network activity^40^ or reduced amygdalae responses to happy, fearful and neutral faces;^41^ this reduction in activity to faces was also seen in a paediatric group^42^. These latter two studies had very small sample sizes (*n* = 10 and 12, respectively), and were likely underpowered. We anticipate, that of the three NDD groups, the OCD would show the fewest differences from the TDs in the neural responses to dynamic happy and angry faces.

## ADHD

ADHD is one of the most common paediatric psychiatric disorders^43^. Behavioural and imaging research has focused on the classic indicators of inattention, hyperactivity and impulsivity, yet increasing evidence suggests that ADHD involves social-cognitive and emotional difficulties also^3,44^. Studies have linked emotional impulsiveness and temperamental dysregulation with ADHD symptoms^45,46^. Yuill et al.^47^ found that boys with ADHD performed poorly when matching emotional faces to situations, but performed similarly to controls with a non-face task. Kats-Gold et al.^48^ reported that boys at risk for ADHD had impaired emotional face identification, and this played a significant role in their social functioning and behaviour. When dynamic faces were used, children with ADHD still had lower accuracy in identifying basic emotions^49^. Hence, we expected to find neuroimaging markers of emotional difficulties in children with ADHD; i.e., reduced activity reflecting reduced awareness or salience of emotions to these children.

Thus, the aims of this study were to determine (a) if the processing of emotional faces differs across the three NDDs, and (b) if the neural mechanisms underlying emotional face processing develop differently over childhood in these groups compared to TD children.

## Materials and methods

Children and adolescents (*n* = 279, 5–19 year olds) were included in the current study (128 ASD, 54 ADHD, 43 OCD and 54 TD). Children were recruited through the Province of Ontario Neurodevelopmental Disorders (POND) network. The children with NDDs were assessed clinically and diagnosed with one of the primary clinical diagnoses. The presence of co-morbidities and the use of psychotropic medication were noted in the participants, but none were excluded on this basis (see Supplemental Information and Supplemental Tables 1 and 2 for further details).

### fMRI Paradigm

The fMRI stimuli consisted of dynamic faces (neutral-to-happy or angry) and dynamic flowers (closed-to-open). Static images of faces (the same faces, neutral and happy and neutral and angry) were taken from the MacBrain Face Stimulus Set^1^ and made dynamic (morphing from neutral to either happy or angry) using Win Morph software. Nature videos of flowers opening and closing in grayscale were used as the non-face stimuli, as detailed in Arsalidou et al.^22^ These stimuli were organized into blocks (13.5 s) of nine trials where the dynamic image was displayed for 480 ms before being replaced by a fixation cross for 1020 ms. Within every block, one of the nine trials was a vigilance trial consisting of a blue star to which the children pressed a button. Each run consisted of 18 pseudo-randomized blocks (six each of happy, angry, flowers) with a 27 s fixation rest period at the halfway point. The stimuli were displayed using Presentation (Neurobehavioral Systems Inc.) software on MR-compatible goggles, and participants were instructed to fixate on the stimuli and respond to the vigilance trials using a dual button MR-compatible keypad. Anatomical images were acquired along with the functional images. Details on the imaging protocols can be found in the Supplemental Information.

### Preprocessing

Image preprocessing of functional data used a combination of AFNI^50,51^ and FSL^52,53^ tools. Slice-timing and motion correction were performed, and the six motion parameters were estimated, from which framewise displacement (FD) was calculated^54^. Volumes with FD > 0.9mm^55^ were censored; participants with more than one third of their volumes censored were excluded from the analyses. Data were smoothed (6 mm FWHM Gaussian kernel), intensity-normalized, and temporally filtered (0.01–0.2 Hz). Signal contributions from the white matter, CSF, whole-brain and six motion parameters were regressed from the data; ICA de-noising was performed via FSL’s FIX.

Preprocessed data were then analyzed with FSL’s FILM^56^. The three dynamic blocks (happy, angry and flowers) were used as explanatory variables and convolved with the hemodynamic response function. The previously estimated six motion parameters and signals from the white matter, CSF and whole brain were used as confound explanatory variables, along with a confound matrix corresponding to the censored volumes to completely remove the effects of the corrupted time points. Within-subject contrasts were generated between each pair of stimuli (happy/angry, happy/flowers, and angry/flowers). Before group-level analyses, images were registered to the Montreal Neurological Institute template using FSL’s Boundary-Based Registration^57^.

### Statistical analysis

Kruskal–Wallis tests were used to compare the median ages and mean FDs in the four diagnostic groups. With significant results, follow-up pairwise comparisons were used and the resulting *p*-values were Bonferroni corrected and significance was held at *p*_corr_ < 0.05. Chi-squared tests were used to determine the presence of proportion differences in sex and acquisition scanner amongst the four diagnostic groups. Upon significance, the Marascuillo procedure^58^ was used to determine which pairwise difference was driving the effect. All tests were run in MATLAB.

For each of the three contrasts, group-level analyses were performed using FSL’s FLAME^59^. Within-group means were determined using one-sample *t*-tests (see Supplemental Information). Differences amongst the four diagnostic groups, along with group-by-age interactions, were investigated using voxelwise *F*-tests. Upon significance of an *F*-test, voxelwise post hoc pairwise *t-*tests between the four diagnostic groups were subsequently run to identify which diagnostic group(s) were driving the significant effect, and in what direction. For all analyses, sex and mean FD were included as covariates, along with a voxelwise covariate modelling the effect of acquisition scanner (see Supplemental Information). Gaussian Random Field theory was used and clusters were determined by *Z* > 2.3, and a cluster-corrected significance threshold of *p*_corr_ < 0.05 was used for the *F*-tests, while cluster significance was held at *p*_corr_ < 0.008 to control for multiple comparisons across the six pairwise comparisons for the *t*-tests.

## Results

After removing subjects who failed to meet the motion criteria, 222 children remained: 49 TD, 87 ASD, 44 ADHD and 42 OCD (Table 1). There was no significant difference in mean FD amongst the four diagnostic groups (*H*(3) = 7.68, *p* = 0.05), nor a significant difference in sex (*Χ*^2^ = 7.14, *p* = 0.07); however, there was a significant difference in age (*H*(3) = 11.75, *p* = 0.01), driven by a difference between the ASD and ADHD participants (see Supplemental Information).

**Table 1:** Participant demographics.

Variable	TD	ASD	ADHD	OCD
*N*	49	87	44	42
Median age (years, ±std.)	11.00 ± 3.59	12.97 ± 3.34	11.06 ± 2.32	12.46 ± 2.59
Sex (M:F)	31:18	71:16	32:12	27:15
Scanner (PrismaFIT:Trio)	16:33	2:85	0:44	0:42
Median mean FD (mm, ±std.)	0.14 ± 0.07	0.20 ± 0.09	0.23 ± 0.10	0.17 ± 0.09

*TD* typical developing, *ASD* autism spectrum disorder, *ADHD* attention deficit hyperactivity disorder, *OCD* obsessive-compulsive disorder, *N* sample size, *M* males, *F* females, *FD* framewise displacement.

**Table 2:** Brain regions showing significant between-group differences in the happy > flowers contrast as shown by an *F* test (*Z* > 2.3, *p*_corr_ < 0.05), along with the significant post hoc pairwise *t* test (*Z* > 2.3, *p*_corr_ < 0.008) results.

Contrast	Cluster	*N*_voxels_	Cluster *p*-value	Max *Z*	Max *Z* coordinates (*x*, *y*, *z*) (mm)	AAL regions (% volume of cluster)
*F* test	1	2297	1.19e−7	4.41	(−24, −64, −4)	MOG.L (30%), LING.L (22%), IOG.L (16%), FFG.L (16%)
	2	2289	1.19e−7	4.73	(22, −54, −6)	MOG.R (26%), FFG.R (17%), LING.R (16%), IOG.R (10%)
TD > ADHD	1	957	7.95e−4	4.38	(−12, −1,6 −6)	ROL.L (28%), INS.L (23%), THA.L (17%)
TD < ASD	1	1728	3.46e−6	4.51	(22, −52, −8)	**MOG.R** (25%), **LING.R** (24%), **FFG.R** (20%), **IOG.R** (12%)
	2	1346	4.46e−5	4.44	(−24, −78, −34)	**MOG.L** (31%), **LING.L** (22%), **FFG.L** (12%)
TD < ADHD	1	1200	1.27e−4	4.19	(−18, −98, −12)	**MOG.L** (39%), **LING.L** (15%), **IOG.L** (13%)
	2	1148	1.86e−4	3.71	(44, −84, 8)	**MOG.R** (34%), **LING.R** (16%), **FFG.R** (14%), **IOG.R** (13%)
TD < OCD	1	11496	1.28e−24	5.23	(24, −54, −10)	**MOG.L** (17%), **MOG.R** (11%)
OCD > ASD	1	1180	1.47e + 4	3.80	(−38, −70, 14)	**MOG.L** (31%), **IOG.L** (27%), **LING.L** (17%), **FFG.L** (11%)
	2	1137	2.01e−4	4.06	(56, −64, −2)	MTG.R (37%), ITG.R (24%), **IOG.R** (21%), **MOG.R** (12%)
OCD > ADHD	1	2001	5.96e−7	4.52	(58, −26, 58)	PoCG.R (49%), SMG.R (33%)

Regions corresponding to the significant between-group difference *F* test results are bolded.

*N*_*voxels*_ number of voxels, *Z* z-statistic, *AAL* automated anatomical labelling, *L* left hemisphere, *R* right hemisphere, *MOG* middle occipital gyrus, *LING* lingual gyrus, *IOG* inferior occipital gyrus, *FFG* fusiform gyrus, *ROL* rolandic operculum, *INS* insula, *THA* thalamus, *MTG* middle temporal gyrus, *ITG* inferior temporal gyrus, *PoCG* postcentral gyrus, *SMG* supramarginal gyrus.

**Fig. 1:**
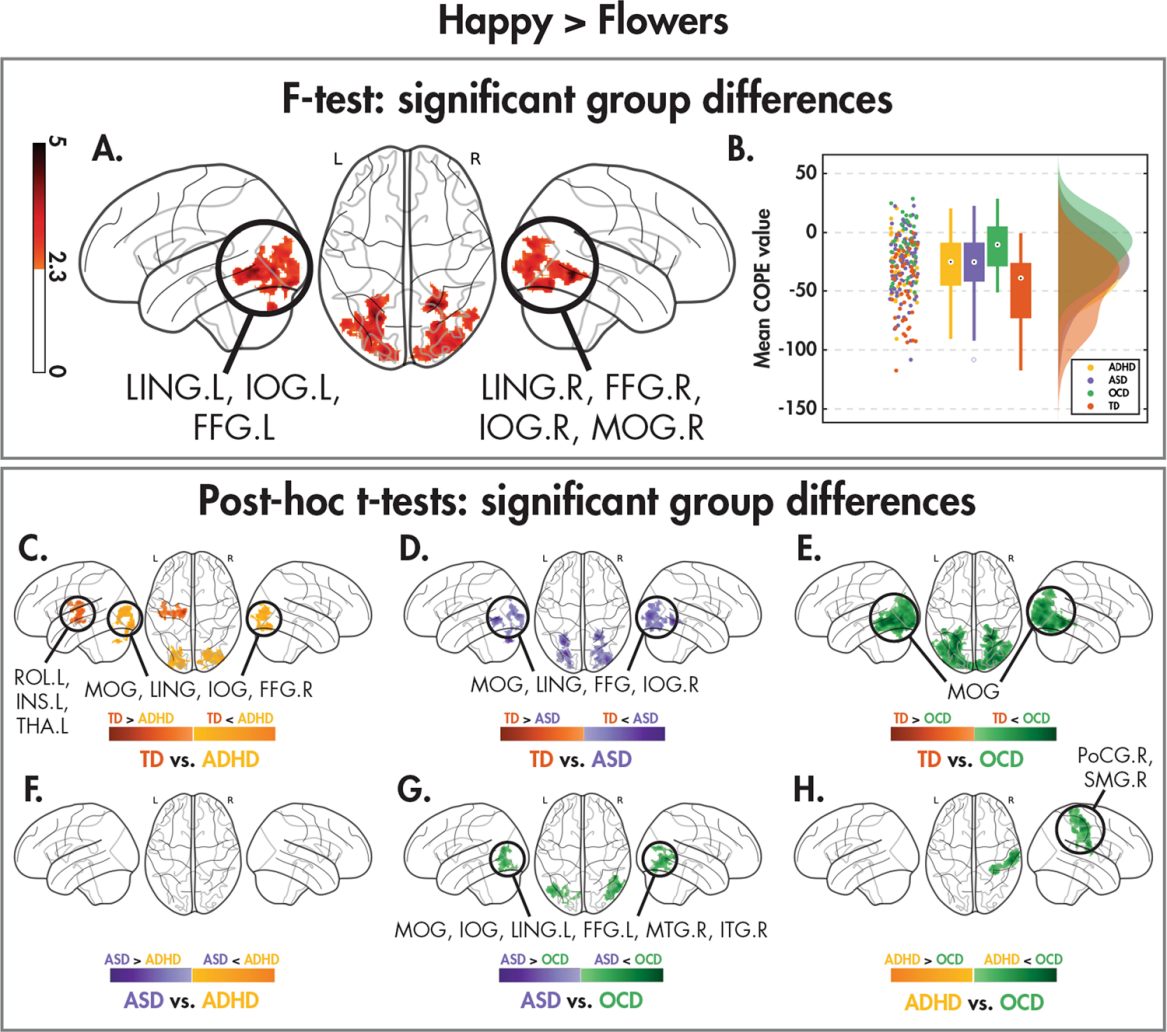
Group differences to dynamic happy faces vs. flowers. Significant (Z > 2.3, *p*_corr_ < 0.05) between-group differences in the happy > flowers contrast as revealed by one-tailed *F* test (**A**), the distribution of the effect across groups (**B**) along with the post hoc pairwise *t* tests (*Z* > 2.3, *p*_corr_ < 0.008) between the four diagnostic groups (**C**, **D** and **E** contrasts between the TDs and the three NDDs and **F**, **G** and **H** the contrasts between the NDDs).

**Fig. 2:**
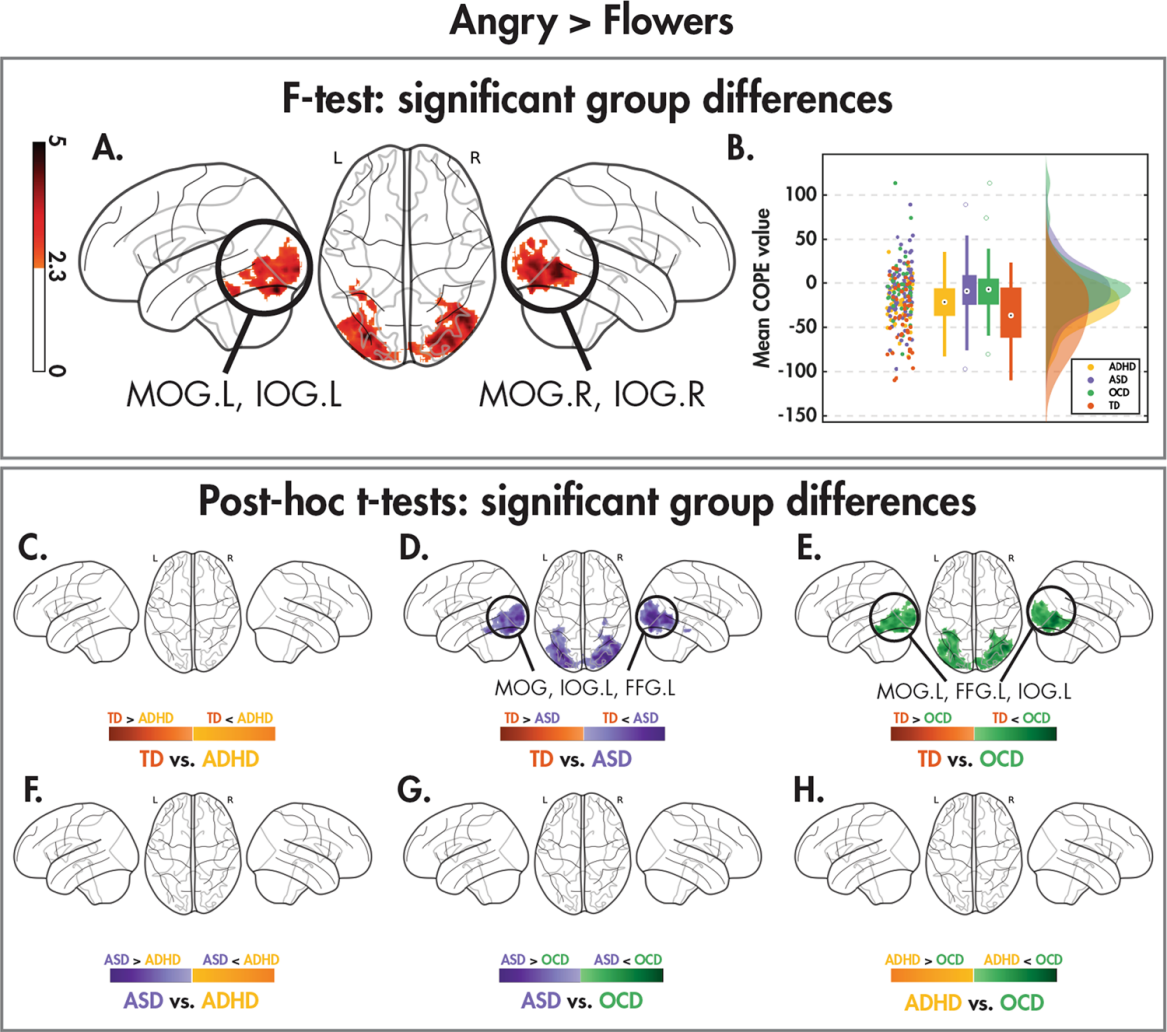
Group differences to dynamic angry faces vs. flowers. Significant (*Z* > 2.3, *p*_corr_ < 0.05) between-group differences in the angry > flowers contrast as shown by one-tailed *F* test, along with the post hoc pairwise *t* tests (*Z* > 2.3, *p*_corr_ < 0.008) between the four diagnostic groups, as in Fig. 1.

**Fig. 3:**
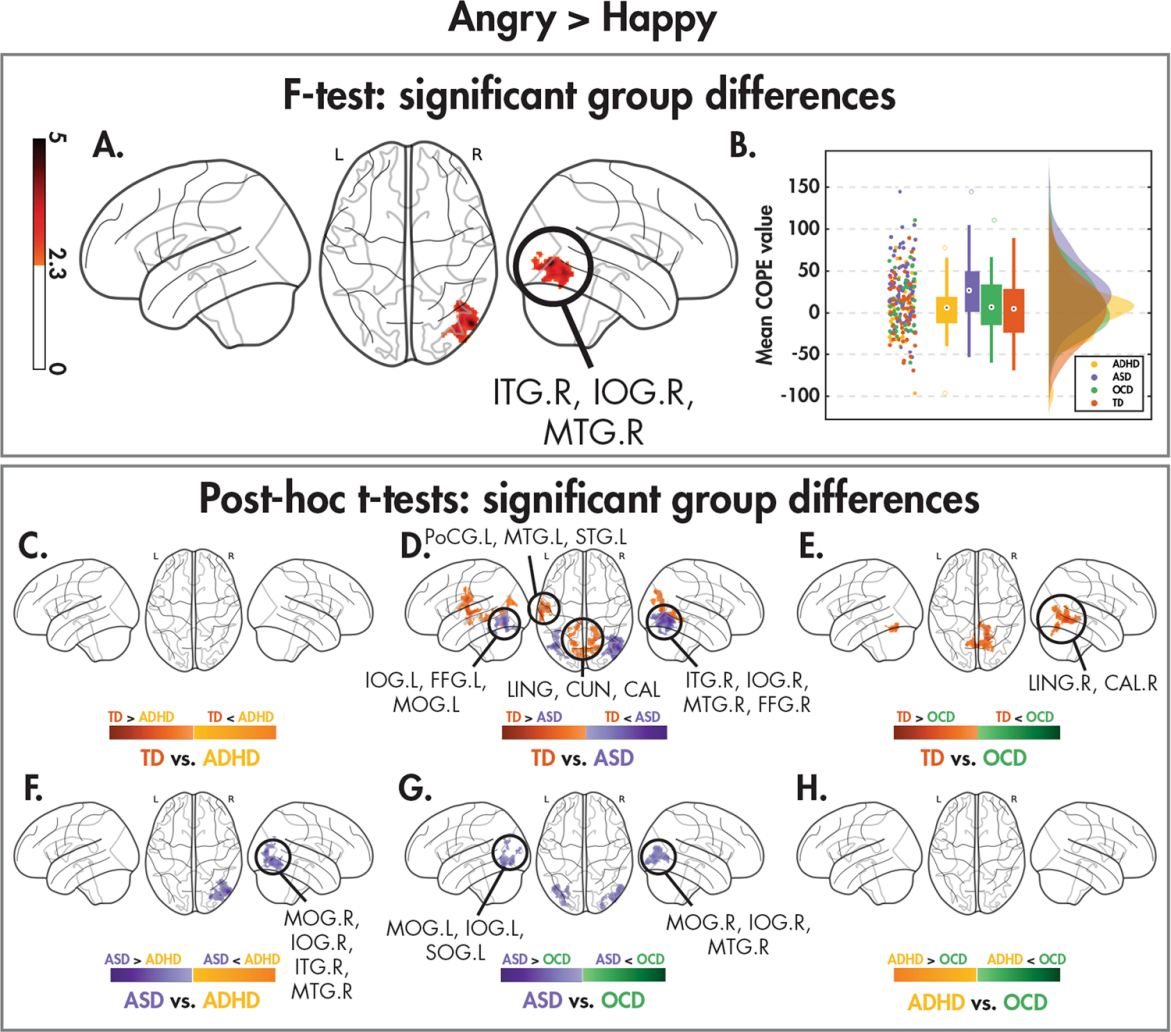
Group differences between dynamic angry and happy faces. Significant (*Z* > 2.3, *p*_corr_ < 0.05) between-group differences in the angry > happy contrast as revealed by one-tailed *F* test, along with the post hoc pairwise *t* tests (*Z* > 2.3, *p*_corr_ < 0.008) between the four diagnostic groups, as in Fig. 1.

**Fig. 4:**
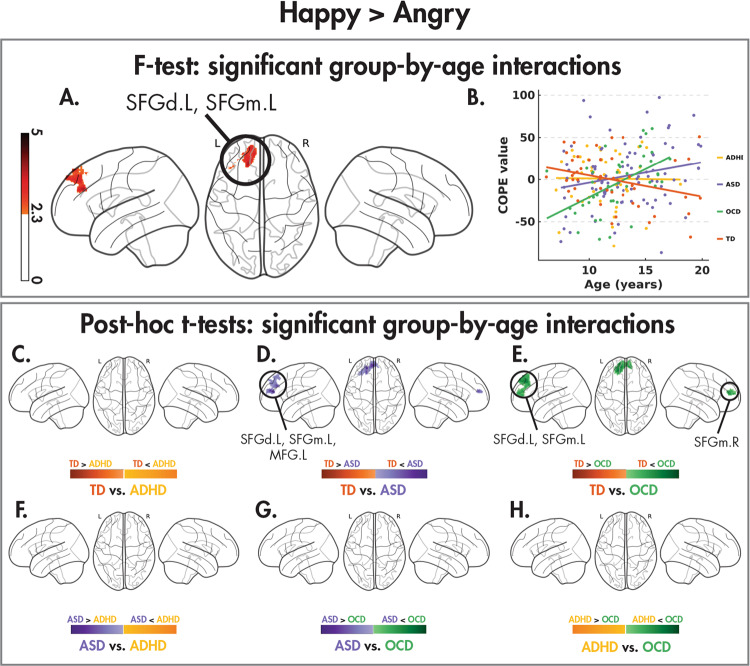
Group-by age-interactions between dynamic happy and angry faces. Significant (*Z* > 2.3, *p*_corr_ < 0.05) group-age interactions in the happy > angry contrast revealed by the one-tailed *F* test, the age by group interaction (**B**), and the post hoc pairwise *t* tests (*Z* > 2.3, *p*_corr_ < 0.008) between the four diagnostic groups.

The voxelwise *F*-tests revealed a similar effect of the diagnostic group when comparing both happy (Fig. 1) and angry (Fig. 2) faces to flowers, which, as revealed by the post hoc *t*-tests, were being driven by differences between the TD and NDD children. A significant effect of diagnostic group was also present when comparing emotion (Fig. 3), caused by differences between the ASD and remaining groups. A significant group-by-age interaction in the emotion contrast was also found (Fig. 4), which was driven by differences in age-related changes between TD and NDD children. A description of these results are detailed in the following paragraphs. Within-group effects can be found in the Supplemental Information, Supplemental Fig. 1, and Supplemental Tables 3–6.

For between-group differences in the happy versus flower contrast, the *F*-test showed significance in the bilateral lingual, inferior occipital and fusiform gyri, and right middle occipital gyrus (Fig. 1A; Table 2). Figure 1B shows that activation to happy faces compared to flowers in these visual regions is *reduced* across all groups (mean COPE values are *negative*). The post hoc pairwise *t*-tests (Fig. 1C–H; Table 2) demonstrated that the significant reduction in activation was driven by differences between the TD and NDD children. In occipital regions, TDs had greater decrease in activation to happy faces compared to flowers than the three NDD groups (Fig. 1C–E) and, within the NDD groups, the OCD children showed increased activation compared to the ASD and ADHD children, in visual and right parietal areas, respectively.

In the angry versus flower contrast, a comparable pattern was seen with the *F*-test, with significant between-group differences occurring in occipital regions including bilateral middle and inferior occipital gyri (Fig. 2A; Table 3). Similarly to happy faces, these regions showed decreased activation to angry faces with respect to flowers (Fig. 2B). The post hoc *t*-tests (Fig. 2C–H; Table 3) confirmed that the differential activation to angry faces versus flowers in TDs was significantly greater than both the ASD and OCD children (but not ADHD), while there were no significant differences amongst the NDD groups. There were no significant group-by-age interactions in either the happy or angry versus flowers contrasts.

**Table 3:** Brain regions showing significant between-group differences in the angry > flowers contrast shown by an *F* test (*Z* > 2.3, *p*_corr_ < 0.05), along with the significant post hoc pairwise *t* test (*Z* > 2.3, *p*_corr_ < 0.008) results.

Contrast	Cluster	*N*_voxels_	Cluster *p*-value	Max *Z*	Max *Z* coordinates (*x*, *y*, *z*) (mm)	AAL regions (% volume of cluster)
*F* test	1	5007	1.34e−13	4.81	(−30, −82, −20)	MOG.L (19%), IOG.L (12%) MOG.R (12%), IOG.R (11%)
TD < ASD	1	8267	2.03e−19	5.49	(−32, −82, −20)	**MOG.L** (18%), **MOG.R** (11.8%), **IOG.L** (11%), FFG.L (10%)
TD < OCD	1	7897	8.38e−19	4.7	(48, −68, −6)	**MOG.L** (16%), FFG.L (12%), **IOG.L** (11%)

Regions corresponding to the significant between-group difference *F* test results are bolded.

*N*_*voxels*_ number of voxels, *Z* z-statistic, *AAL* automated anatomical labelling, *L* left hemisphere, *R* right hemisphere, *MOG* middle occipital gyrus, *IOG* inferior occipital gyrus, *FFG* fusiform gyrus.

More interesting for our questions of emotional face processing, were the between-emotion contrasts. For the angry versus happy faces between-group contrast, significant *F*-test differences were found in a small cluster straddling the right inferior occipital, inferior and middle temporal gyri (Fig. 3A, B; Table 4). The post hoc *t*-tests (Fig. 3C–H; Table 4) revealed that this cluster was significant in the ASD pairwise comparisons, with ASD children showing increased activation to angry compared to happy faces compared to all three of the other groups, who showed minimal difference between the two emotions. The TD children, however, showed greater activation than the ASD to angry than happy faces in the cuneus and occipital areas bilaterally and in left temporal regions (Fig. 3D). The TD also showed greater activity to angry than happy faces than the OCD in occipital-temporal areas (Fig. 3E), while there were no differences between the ADHD and TD in this contrast (Fig. 3C).

**Table 4:** Brain regions showing significant between-group differences in the angry > happy contrast shown by an *F* test (*Z* > 2.3, *p*_corr_ < 0.05), along with the significant post hoc pairwise *t* test (*Z* > 2.3, *p*_corr_ < 0.008) results.

Contrast	Cluster	*N*_voxels_	Cluster *p*-value	Max *Z*	Max *Z* coordinates (*x*, *y*, *z*) (mm)	AAL regions (% volume of cluster)
*F* test	1	810	2.96e−3	4.63	(52, −72, 0)	ITG.R (37%), IOG.R (31%), MTG.R (20%)
TD > ASD	1	1674	6.20e−6	3.71	(18, −82, 34)	LING.R (19%), CUN.R (15%), CAL.R (13%), LING.L (12%), CAL.L (12%), CUN.L (12%)
	2	753	4.73e−3	4.26	(−52, −14, 24)	PoCG.L (56%), MTG.L (23%), STG.L (15%)
TD > OCD	1	1255	1.02e−4	4.00	(16, −54, −6)	LING.R (36%), CAL.R (17%)
TD < ASD	1	1570	1.19e−5	4.58	(52, −72, 0)	**ITG.R** (30%), **IOG.R** (27%), **MTG.R** (17%), FFG.R (14%)
	2	756	4.62e−3	3.97	(−44, −68, −14)	IOG.L (32%), FFG.L (24%), MOG.L (17.3%)
ASD > ADHD	1	1137	2.38e−4	4.58	(54, −74, −2)	MOG.R (28%), **IOG.R** (26%), **ITG.R** (24%), **MTG.R** (12%)
ASD > OCD	1	821	2.7e−3	3.51	(52, −72, 0)	MOG.R (36%), **IOG.R** (27%), **MTG.R** (26%)
	2	707	6.98e−3	3.87	(−32, −78, −8)	MOG.L (48%), IOG.L (34%), SOG.L (10%)

Regions corresponding to the significant between-group difference *F* test results are bolded.

*N*_*voxels*_ number of voxels, *Z* z-statistic, *AAL* automated anatomical labelling, *L* left hemisphere, *R* right hemisphere, *ITG* inferior temporal gyrus, *IOG* inferior occipital gyrus, *MTG* middle temporal gyrus, *ITG* inferior temporal gyrus, *LING* lingual gyrus, *CUN* cuneus, *CAL* calcarine sulcus, *PoCG* postcentral gyrus, *STG* superior temporal gyrus, *FFG* fusiform gyrus, *MOG* middle occipital gyrus, *SOG* superior occipital gyrus.

Finally, in happy versus angry faces, the only significant group-by-age interaction was found: increased activity in the left superior and medial frontal gyri (Fig. 4A, B; Table 5), which was driven by differences in age-related changes between TD and both the ASD and OCD children (Fig. 4D–E; Table 5). The ASD and OCD groups recruited these frontal regions less when processing happy compared to angry faces during childhood but more in adolescence, while the TD children showed the opposite effect; the ADHD children showed no activation difference between happy and angry faces in this region, and this remained constant over age. There were no differences amongst the NDD groups in this contrast (Fig. 4F–G).

**Table 5:** Brain regions showing significant group-by-age interactions in the happy > angry contrast shown by an *F* test (*Z* > 2.3, *p*_corr_ < 0.05), along with the significant post hoc pairwise *t* test (*Z* > 2.3, *p*_corr_ < 0.008) results.

Contrast	Cluster	*N*_voxels_	Cluster *p*-value	Max *Z*	Max *Z* coordinates (*x*, *y*, *z*) (mm)	AAL regions (% volume of cluster)
Happy > angry	1	612	0.02	4.11	(−8, 52, 54)	SFGd.L (81%), SFGm.L (17%)
TD < ASD	1	1331	6.87e−5	5.01	(−18, 48, 16)	**SFGd.L** (41%), **SFGm.L** (35%), MFG.L (14%)
TD < OCD	1	1975	9.54e−7	4.61	(−8, 52, 54)	**SFGd.L** (41%), **SFGm.L** (38%), SFGm.R (15%)

Regions corresponding to the significant between-group difference *F* test results are bolded.

*N*_*voxels*_ number of voxels, *Z* z-statistic, *AAL* automated anatomical labelling, *L* left hemisphere, *SFGd* superior frontal gyrus, *SFGm* medial frontal gyrus, *MFG* middle frontal gyrus.

The within group means for each of the four groups for these four contrasts are shown in Supplemental Fig. 1.

## Discussion

Using dynamic happy and angry faces, we demonstrated that the children with NDDs shared neural processing of emotional faces, seen particularly in occipital and temporal regions, compared to their TD peers. Although these patterns varied with the emotion expressed, our findings suggest similar neural mechanisms underlying socio-emotional difficulties across NDDs. Importantly, in the contrast between happy and angry faces, there was an age-by-group interaction that involved the left superior frontal gyrus, indicating different developmental trajectories of brain areas engaged in emotional face processing, a widening gap between NDDs and TD and increasing difficulties in the NDDs with age. These findings are discussed below, in relation to emotional processing in these child psychiatric populations.

Shared alterations in medial and lateral occipital activity between dynamic faces and dynamic flowers in the NDDs may reflect their shared difficulties in emotional face recognition. The TD group had a greater decrease in activation to faces compared to flowers than the NDD group, which suggests more distinctive processing of the dynamic facial stimuli by the TD; in contrast, the NDDs showed more similar processing of the dynamic stimuli, regardless of whether they were faces or flowers. Face recognition is subserved by a distributed brain network that engages in processing and integrating visual information and the inferior occipital and fusiform regions have key roles in this function. This network is present at birth and matures by late adolescence and adulthood^60^, with maturation facilitating efficiency in the speed and accuracy of face processing^61^. Greater decreases to faces than flowers in the TD group may reflect a more mature face network in the TD youth such that less effort was needed in face processing but greater activation was induced to the novelty of dynamic flowers. Atypical salience processing of both the novel dynamic flowers and the socially salient faces may be common across NDDs. Under-connectivity in the salience and visual networks has frequently been shown in ASD^62,63^, but our findings suggest the ADHD and OCD groups also demonstrate alterations in salience processing, and that this effect was greatest for the OCD group.

Impaired emotional processing has been implicated in OCD, particularly with aversive emotions, such as disgust. Although atypical involvement of limbic areas^41,42^ and fronto-striatal circuits has been reported in emotional processing in OCD, a recent meta-analysis of 25 neuroimaging studies reported an expanded brain network including the middle temporal and inferior occipital regions in OCD. In this network context, limbic hyperactivation influences early recruitment in the occipital region to visual stimuli, and this is then linked to upregulated amygdala activity^64^. In addition, a study investigating metabolic activity also suggested visual processing deficits in OCD^65^. Our OCD cohort showed the least neural differentiation between faces and flowers which may reflect generally reduced activation in processing visual objects, regardless of stimuli or emotional valence in an emotional context. For the OCD-ADHD contrast, greater activity in the right postcentral/supramarginal gyri was seen in the OCD—an area involved in emotional understanding, including egocentricity of emotions^66^, suggesting that compared to ADHD, they were engaging these regions appropriately for dynamic face stimuli. Others have also found this ability intact in ASD;^67^ thus, these data suggest the difficulty of egocentricity in emotional perception is more prominent in youth with ADHD.

Although both happy and angry faces, contrasted to dynamic flowers, demonstrated comparable patterns across participants, the decreased occipital activity was less marked to angry than happy faces in TD, indicating that dynamic angry faces are more salient visual stimuli and require more effort for processing even in TD^68^. This is consistent with the asynchronous maturation of emotional face recognition, where it is later for angry than happy faces^69^. This neural differentiation between angry faces versus flowers shown in TD, however, was not seen in children with ASD or OCD. This may reflect the difficulties that those with ASD have with angry expressions (negative emotions) from infancy to adulthood^70^, as well as those with OCD have with negative emotions, including anger^37,64^. Although challenges in facial emotion recognition in ASD have been observed in both positive and negative emotions, children with ASD generally show worse performance for negative emotions^71–73^. Our study also supported greater impairment in processing negative emotions in ASD in the analysis contrasting the emotional faces.

Angry faces led to greater activation in the right inferior occipital and middle and inferior temporal areas, consistent with studies that show greater visual activity to negative than positive faces^68,74^. Interestingly, the group differences were driven by the ASD who showed reduced activity in the cuneus and occipital area bilaterally and in left superior and middle temporal regions compared to TDs. The superior and middle temporal regions are closely linked with a biological motion, including facial movements^21^. Given that the expression of anger is usually less common than happy expressions in everyday life, we suggest that the TD group are attending more to angry than happy faces. The ASD group recruited these areas less than their TD peers, yet showed greater activity than TD, ADHD and OCD in primary face processing regions (right middle, inferior temporal and inferior occipital-fusiform regions), suggesting greater visual salience for angry than happy faces. The increased activity in TD compared to ASD and OCD youth (Fig. 3D–E) was see in more lateral, temporal, medial posterior regions, suggesting higher-order processing areas were recruited by the TD.

The only age-related changes were seen with the happy > angry faces contrast in the left superior frontal gyrus. While activation of this region decreased with age in TD children, it increased for ASD and OCD and showed no age effects in ADHD. Thus, TD children recruited the left superior frontal gyrus more for angry faces in adolescence, while the OCD and ASD recruited it more for happy faces in adolescence. Previous literature has shown that the maturation in prefrontal regions supports the detection and evaluation of angry faces in the TD population^75^. Superior frontal gyrus activation in emotional face processing was reported across studies in a meta-analysis^76^ and the left superior frontal gyrus was associated with cognitive activities including processing pleasant and unpleasant emotions^77^, self-criticism^78^ and attention to negative emotions^79^. The ability to recognise and interpret emotions matures from early childhood through adolescence^80,81^ and frontal engagement would be refined with age, particularly for the emotions which are experienced less frequently. Happiness is the only one of the six basic emotions that is definitely positive and is the first emotion to be accurately identified in early development^82^. Studies have reported typical processing of happy effect in ASD, which was interpreted as due to greater familiarity with happy faces^83^. Our results, however, showed processing of happy effect requiring more frontal engagement with age in ASD and OCD; thus, even though they may understand and respond appropriately to happy faces, processing them may still remain difficult. This finding is supported by a meta-analytic review for facial emotion recognition in ASD that showed that difficulties increase with age in recognising happiness but not anger^84^. This suggests that the recognition for happy faces improves with age in TDs, but not in ASD. In addition, the ability for those with ASD to recognize negative emotions such as anger may lag behind their TD peers. The finding from the OCD group is of particular interest, suggesting that they experience as much difficulty in processing positive emotions as the ASD group, and it may also worsen with age. Only the ADHD group showed no age-related effects. As there was a significant difference in age between the ASD and ADHD children, however, the lack of group-by-age interaction between these two groups should be interpreted cautiously.

In summary, the present study demonstrated that NDD youth shared alterations in processing dynamic emotional faces: a similar functional mechanism was engaged for both dynamic faces and flowers. This suggests that the emotional difficulties shared by NDDs may be partly attributed to atypical visual information processing interfering with social-emotional information management. Although these patterns were similar across the NDDs, the OCD group showed the least differentiation between faces and flowers and between happy and angry faces, which may be attributed to reduced visual processing in OCD, related to hyperactivity in fronto-striatal circuit. In addition, those with OCD required greater engagement of frontal regions to process emotions that increased with age. Contrary to our expectations, OCDs exhibited the least differentiation of the dynamic visual stimuli, and shared increased difficulties with age for happy faces with the ASD group.

The youth with ASD demonstrated more marked impairments in processing negative emotion and the negative-emotion-related activity in temporo-occipital regions was unique to the ASD group. As with the OCD youth, the ASD group also showed increased frontal involvement with age to happy faces. Together these findings indicate that although happy faces are recognisable in ASD, frontal engagement does not decline with age, suggesting that the emotional processing requires similar (or increased) efforts at a neural level, contributing to their life-long difficulties. In contrast, brain responses to angry faces were indistinguishable from the dynamic flowers in ASD and OCD youth, suggesting that angry faces were treated simply as visual stimuli. The ADHD group showed the least impairment across all contrasts in our study. Thus, although all three diagnostic groups shared some alterations in dynamic face processing, there were also some distinct patterns reflecting specific aspects of emotional processing within each disorder.

The significant overlap we report across disorders supports a growing literature that suggests that the neurobiological susceptibility in NDDs needs understanding beyond traditional diagnosis-based categories. Alternative approaches, such as subgrouping by data-driven factors based on dimensional measures have been attempted, but to subgroup NDDs into neurobiologically homogeneous groups is still very challenging. A deep-and-big data approach considering multiple dimensions, and their interactions, engaged in human cognitive processes may be necessary to understand shared neurobiology.

Lastly, we note that the primary diagnosis in NDDs was accounted for in the current study and the Supplementary information revealed a significant portion of children have a co-occurring diagnosis. Thus, the results and discussion should be considered in light of this further evidence of overlap in the NDDs. The purpose of this study, however, was to determine if shared mechanisms underlay socio-emotional difficulties commonly existing in the NDDs. Our results demonstrated that all three NDD groups shared alterations in processing dynamic emotional faces compared to their TD peers, and suggested that these groups of children need to be investigated together in a single cohort to identify biologically homogeneous groups above and beyond their diagnoses.

## Supplementary information

Supplemental Information
