# Supplementary material for: Emotional face processing across neurodevelopmental disorders: a dynamic faces study in children with autism spectrum disorder, attention deficit hyperactivity disorder and obsessive-compulsive disorder
Source: Transl Psychiatry. 2020 Nov 2;10:375. doi: 10.1038/s41398-020-01063-2 (PMC7608673; doi:10.1038/s41398-020-01063-2)
Supplement: Supplementary file 1 — Supplemental Information [file 41398_2020_1063_MOESM1_ESM.docx]

**Emotional face processing across neurodevelopmental disorders:**

**A dynamic faces study in children with autism spectrum disorder, attention deficit hyperactivity disorder and obsessive compulsive disorder**

**Supplemental methods**

**Participants**

The Province of Ontario Neurodevelopmental Disorders (POND) network is a research collaboration between five sites (Holland Bloorview Kids Rehabilitation Hospital, Toronto; The Hospital for Sick Children, Toronto; McMaster Children’s Hospital, Hamilton; and Lawson Health Research Institute, London; Queen’s University, Kingston), and aims to understand the neurobiology, genetic factors and environmental factors underpinning neurodevelopmental disorders (see pond-network.ca for more information). For this study, participants with neurodevelopmental disorders were recruited through POND who had a primary diagnosis of ASD, ADHD or OCD, and TD participants with the absence of prematurity and neurodevelopmental, psychiatric and neurological diagnoses were recruited through advertising. Both sets of participants were required to have sufficient English skills to complete behavioural assessments. Approval for this study was approved by each institution’s respective research ethics boards, and written informed consent or assent was obtained from the primary caregiver or participant where appropriate.

Disorder-specific diagnostic assessments were performed in all participants to confirm clinical diagnoses with expert clinical judgement: the Autism Diagnostic Observation Schedule-2 (ADOS)^1^ and Autism Diagnostic Interview-Revised (ADI-R)^2^ for ASD; the Kiddie Schedule for Affective Disorders and Schizophrenia (K-SADS)^3^ and Parent Interview for Child Symptoms (PICS)^4^ for ADHD and the K-SADS and Children’s Yale-Brown Obsessive Compulsive Scale (CY-BOCS)^5^ for OCD. IQ was assessed in all participants with the Wechsler Abbreviated Scale of Intelligence (WASI), Wechsler Intelligence Scale for Children-IV, or Stanford Binet Intelligence Scales (SB).

The presence of co-morbidities and use of psychotropic medication were noted, but participants were not excluded on this basis. Psychiatric comorbidities were assessed using the Diagnostic and Statistical Manual of Mental Disorders IV^6^ or V^7^ on 165 of the 222 participants included in the final analyses while psychotropic medications were recorded for 192 of the 222 participants included in the final analyses; further information can be found in Supplemental Tables 1 and 2, respectively.

**Image acquisition**

Imaging was performed on either a 3T Siemens MAGNETOM Trio MRI scanner with a 12-channel head coil (N=259) or a 3T Siemens MAGNETOM PrismaFIT MRI scanner with a 20 channel head and neck coil (N=20). Anatomical scans were acquired using a 3D T1-weighted MPRAGE sequence on both the Trio (TR/TE/TI=2300/2.96/900ms; FA=9°; FOV=240x256mm; 192 slices; 1mm isotropic resolution; 5:03min scan time) and PrismaFIT (TR/TE/TI=1870/3.14/945ms; FA=9°; FOV=240x256mm; 192 slices; 0.8mm isotropic resolution; scan time 5:10min). Functional images were acquired with single-shot echo planar imaging sequence with the same protocol on the Trio and PrismaFIT (TR/TE=2000/30ms; FA=70°; FOV=192x192mm; 30 slices; resolution=3x3x5mm; 4:46min scan time). Visual stimuli for the functional task were displayed on MR-compatible goggles. Children responded to vigilance trials using an MR-compatible keypad. Stimuli were displayed and performance was recorded using *Presentation* (Neurobehavioral Systems Inc.).

**Analyses**

There was a difference in acquisition scanner (*Χ*^2^ = 51.15, *p* < 0.01), and the Marascuillo procedure identified that, at *p* < 0.05, the TD group had a significantly higher proportion of children scanned on the PrismaFIT compared to the ASD, ADHD and OCD children. Due to the unequal distribution of the TD and NDD children across the two scanners, the significant effect of the PrismaFIT scanner was estimated within the TD group in a separate analysis using FLAME, and used as a voxelwise covariate. For all tests, the Automated Anatomical Labelling (AAL)^8^ atlas was used to determine the anatomical location of significant clusters in the cerebral cortex. For each cluster, AAL regions which comprised at least 10% of the cluster’s volume were reported.

**Supplemental results**

There was a significant difference in age among the four diagnostic groups (*H*(3)=11.75, *p*=0.01). Follow-up pairwise comparisons with adjusted *p*-values showed there were no significant differences in age between the TD and ASD (*H*(1) = 4.09, p_corr_ = 0.26), ADHD (*H*(1) = 0.07, p_corr_ = 1.00) or OCD (*H*(1) = 2.24, p_corr_ = 0.81) children, nor between the OCD and ASD (*H*(1) = 0.71, p_corr_ = 1.00) or ADHD (*H*(1) = 5.56, p_corr_ = 0.11) children; however, there was a difference in age between the ASD and ADHD (*H*(1) = 10.00, p_corr_ = 0.01) children. Age was used as a covariate in all subsequent analyses to account for this difference.

The significant within-group means for the happy>flowers, angry>flowers, happy>angry, and angry>happy contrasts are presented in Supplemental Figure 1 and Supplemental Information Tables 3–6. Overall, the TD children demonstrated different activity patterns across the four contrasts, but these patterns were more limited and differed across the NDD groups. Consistent with the previous literature, dynamic faces activated a variety of regions including frontal, motor and subcortical regions, and areas engaged in emotional information processing in TDs (Fig. 1); happy faces exhibited greater activation than angry faces in the left middle frontal gyrus and putamen, while angry>happy faces showed increases in right occipital and parahippocampal regions. These same contrasts in ASD youth showed left frontal areas engaged preferentially in processing happy faces and large posterior occipital areas bilaterally active to angry faces. In contrast, the ADHD and OCD groups showed no differences between the happy and angry faces, only between the flowers and faces (Supplemental Fig. 1).

**Supplemental Figures**


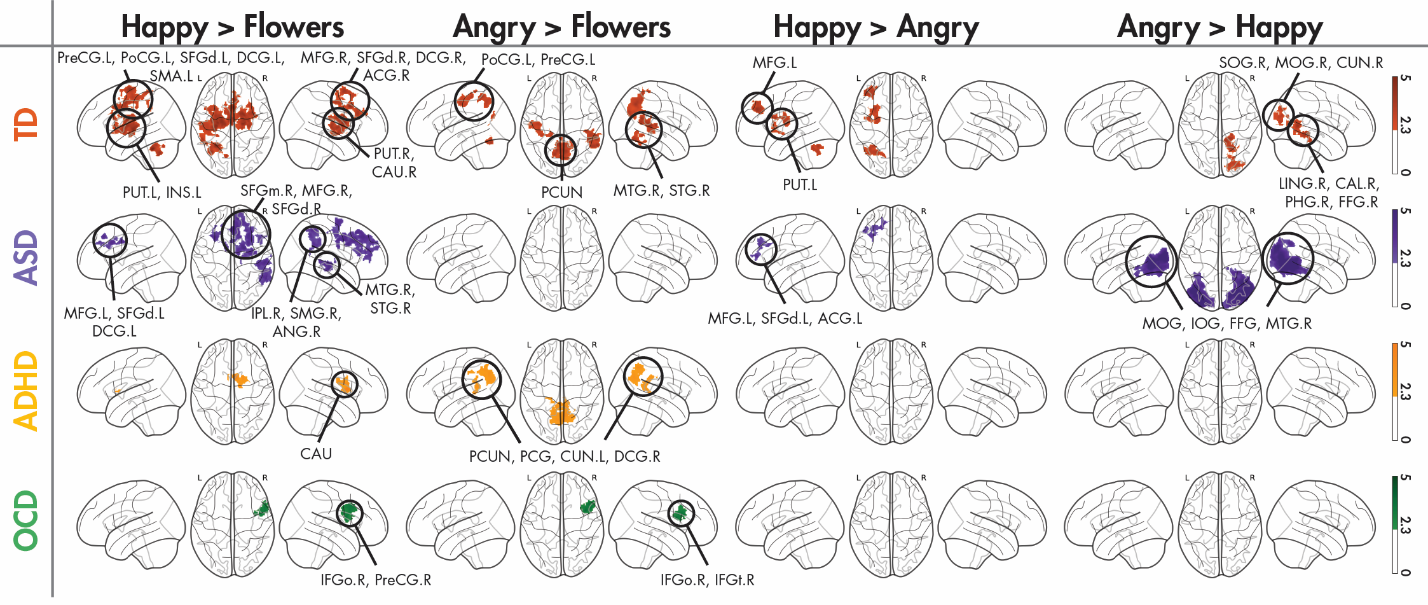


**Supplemental Figure 1**: Significant (Z > 2.3, *p*_corr_ < 0.05) within-group means for the TD, ASD, ADHD, and OCD children in the happy > flowers, angry > flowers, happy > angry, and angry > happy contrasts shown by one-tailed *t* tests.

**Supplemental Tables**

**Supplemental Table 1**: Details on documented psychiatric comorbidities on the final analyzed sample.

| **Variable** | **TD** | **ASD** | **ADHD** | **OCD** |
| --- | --- | --- | --- | --- |
| N | 49 | 31 | 44 | 41 |
| Number of participants with a psychiatric comorbidity | 0 | 21 | 30 | 23 |
| Comorbidity  breakdown | - | 8 ADHD  2 ANX  2 ID  1 LD  2 ADHD + ANX  2 ANX + LD  1 ADHD + ID  3 ADHD + ANX + LD | 14 LD  5 DB  3 ANX  3 ANX + LD  3 LD + DB  1 ANX + DB  1 OCD + LD + DB | 12 ANX  4 ADHD  2 ADHD + ANX  1 ADHD + LD  2 ADHD + ANX + DB  1 ASD + ADHD + ANX  1 ASD + ANX + LD |

TD: typical developing; ASD: autism spectrum disorder; ANX: anxiety disorder; LD: learning disorder; ID: intellectual disability; DB: disruptive behaviour disorder.

**Supplemental Table 2**: Details on documented psychotropic medications on the final analyzed sample.

| **Variable** | **TD** | **ASD** | **ADHD** | **OCD** |
| --- | --- | --- | --- | --- |
| N | 49 | 63 | 41 | 39 |
| Number of participants on psychotropic medication | 0 | 7 | 6 | 15 |
| Psychotropic medication breakdown | - | 1 Stimulant  1 Atomoxetine  1 AAP  1 Stimulant + melatonin  1 Stimulant + SSRI  1 Stimulant + AAP  1 Stimulant + Atomoxetine + AAP | 3 Stimulant  1 Guanfacine  2 Stimulant + AAP | 5 SSRI  1 AAP  1 Anticonvulsant  2 SSRI + AAP  2 Stimulant + SNRI  1 Stimulant + SSRI  1 Stimulant + Clonidine  1 Stimulant + SSRI + Clonidine  1 SSRI + AAP + Anticonvulsant |

TD: typical developing; ASD: autism spectrum disorder; ADHD: attention deficit hyperactivity disorder; OCD: obsessive compulsive disorder; N: sample size; AAP: atypical antipsychotic; SSRI: selective serotonin reuptake inhibitor; SNRI: selective norepinephrine reuptake inhibitor

**Supplemental Table 3**: Brain regions showing significant (Z > 2.3, *p*_corr_ < 0.05) within-group means in the happy > flowers contrast as revealed by one-tailed *t* tests.

| **Contrast** | **Cluster** | **N_voxels_** | **Cluster**  ***p*-value** | **Max**  **Z** | **Max Z**  **coordinates**  **(x, y, z) (mm)** | **AAL regions**  **(% volume of cluster)** |
| --- | --- | --- | --- | --- | --- | --- |
| TD | 1 | 3675 | 6.12e^-11^ | 4.67 | (-28, -18, 0) | PUT.L (25%), PUT.R (14%), INS.L (13%), CAU.R (13%) |
|  | 2 | 1593 | 8.29e^‑6^ | 4.56 | (-24, -12, 68) | PreCG.L (41%), PoCG.L (38%), SFGd.L (11%) |
|  | 3 | 1309 | 5.79e^-5^ | 4.52 | (2, 14, 38) | DCG.L (37%), SMA.L (20%), DCG.R (19%), ACG.R (12%) |
|  | 4 | 611 | 0.01 | 3.92 | (36, 4, 66) | MFG.R (61%), SFGd.R (35%) |
| ASD | 1 | 3850 | 2.56e^-11^ | 4.61 | (20, 2, 38) | SFGm.R (23%), MFG.R (21%), SFGd.R (18%) |
|  | 2 | 1106 | 2.54e^-3^ | 4.16 | (60, -48, 56) | IPL.R (43%), SMG.R (42%), ANG.R (12%) |
|  | 3 | 701 | 6.56e^-3^ | 4.33 | (56, -24, -12) | MTG.R (63%), STG.R (36%) |
|  | 4 | 490 | 0.05 | 3.99 | (-10, 2, 34) | MFG.L (45%), SFGd.L (24%), DCG.L (23%) |
| ADHD | 1 | 558 | 0.02 | 4.75 | (6, 10, 12) | CAU.R (89%), CAU.L (10%) |
| OCD | 1 | 780 | 3.34e^-3^ | 3.62 | (58, 8, 20) | IFGo.R (46%), PreCG.R (42%) |

N_voxels_: number of voxels; Z: z-statistic; AAL: automated anatomical labelling; TD: typical developing; ASD: autism spectrum disorder; ADHD: attention deficit hyperactivity disorder; OCD: obsessive compulsive disorder; L: left hemisphere; R: right hemisphere; PUT: putamen; INS: insula; CAU: caudate; PreCG: precentral gyrus; PoCG: postcentral gyrus; SFGd: superior frontal gyrus; DCG: midcingulate gyrus; SMA; supplemental motor area; ACG: anterior cingulate gyrus; MFG: middle frontal gyrus; SFGm: medial frontal gyrus; IPL: inferior parietal lobule; SMG: supramarginal gyrus; ANG: angular gyrus; MTG: middle temporal gyrus; STG: superior temporal gyrus; IFGo: inferior frontal gyrus, pars opercularis

**Supplemental Table 4**: Brain regions showing significant (Z > 2.3, *p*_corr_ < 0.05) within-group means in the angry > flowers contrast as revealed by one-tailed *t* tests.

| **Contrast** | **Cluster** | **N_voxels_** | **Cluster**  ***p*-value** | **Max**  **Z** | **Max Z**  **coordinates**  **(x, y, z) (mm)** | **AAL regions**  **(% volume of cluster)** |
| --- | --- | --- | --- | --- | --- | --- |
| TD | 1 | 1642 | 6.62e^-6^ | 4.12 | (-6, -50, 56) | PCUN.R (50%), PCUN.L (28%) |
|  | 2 | 1115 | 2.55e^-4^ | 4.55 | (60, -38, 6) | MTG.R (49%), STG.R (47%) |
|  | 3 | 780 | 3.53e^-3^ | 3.43 | (-40, -14, 48) | PoCG.L (89%), PreCG.L (11%) |
| ASD | N.S. | | | | | |
| ADHD | 1 | 1807 | 2.26e^-6^ | 3.77 | (8, -56, 48) | PCUN.R (42%), PCUN.L (39%), CUN.L (11%) |
|  | 2 | 667 | 9.27e^-3^ | 3.66 | (-8, -42, 14) | PCG.R (32%), PCG.L (27%), PCUN.R (21%), DCG.R (14%) |
| OCD | 1 | 628 | 0.01 | 4.13 | (42, 8, 18) | IFGo.R (664%), IFGt.R (29%) |

N_voxels_: number of voxels; Z: z-statistic; AAL: automated anatomical labelling; TD: typical developing; ASD: autism spectrum disorder; ADHD: attention deficit hyperactivity disorder; OCD: obsessive compulsive disorder; L: left hemisphere; R: right hemisphere; PCUN: precuneus, MTG: middle temporal gyrus; STG: superior temporal gyrus; PoCG: postcentral gyrus; PreCG: precentral gyrus; CUN: cuneus; PCG: posterior cingulate gyrus; DCG: midcingulate gyrus; IFGo: inferior frontal gyrus, pars opercularis; IFGt: inferior frontal gyrus, pars triangularis

**Supplemental Table 5**: Brain regions showing significant (Z > 2.3, *p*_corr_ < 0.05) within-group means in the happy > angry contrast as revealed by one-tailed *t* tests.

| **Contrast** | **Cluster** | **N_voxels_** | **Cluster**  ***p*-value** | **Max**  **Z** | **Max Z**  **coordinates**  **(x, y, z) (mm)** | **AAL regions**  **(% volume of cluster)** |
| --- | --- | --- | --- | --- | --- | --- |
| TD | 1 | 731 | 0.01 | 3.84 | (-26, 12, 20) | PUT.L (77%) |
|  | 2 | 583 | 0.02 | 4.27 | (-40, 42, 34) | MFG.L (92%) |
| ASD | 1 | 591 | 0.02 | 3.71 | (-14, 42, 10) | MFG.L (51%), SFGd.L (22%), ACG.L (13%) |
| ADHD | N.S. | | | | | |
| OCD | N.S. | | | | | |

N_voxels_: number of voxels; Z: z-statistic; AAL: automated anatomical labelling; TD: typical developing; ASD: autism spectrum disorder; ADHD: attention deficit hyperactivity disorder; OCD: obsessive compulsive disorder; L: left hemisphere; R: right hemisphere; PUT: putamen; MFG: middle frontal gyrus; SFGd: superior frontal gyrus; ACG: anterior cingulate gyrus

**Supplemental Table 6**: Brain regions showing significant (Z > 2.3, *p*_corr_ < 0.05) within-group means in the angry > happy contrast as revealed by one-tailed *t* tests.

| **Contrast** | **Cluster** | **N_voxels_** | **Cluster**  ***p*-value** | **Max**  **Z** | **Max Z**  **coordinates**  **(x, y, z) (mm)** | **AAL regions**  **(% volume of cluster)** |
| --- | --- | --- | --- | --- | --- | --- |
| TD | 1 | 853 | 2.09e^-3^ | 4.14 | (18, -54, 6) | LING.R (33%), CAL.R (28%), PHG.R (10%), FFG.R (10%) |
|  | 2 | 603 | 0.02 | 3.61 | (24, -72, 22) | SOG.R (47%), MOG.R (34%), CUN.R (17%) |
| ASD | 1 | 6817 | 8.39e^-17^ | 7.15 | (50, -68, 0) | MOG.R (24%), FFG.R (17%), IOG.R (13%), MTG.R (10%) |
|  | 2 | 4574 | 1.25e^-12^ | 6.26 | (-42, -70, -8) | MOG.L (40%), IOG.L (18%), FFG.L (17%) |
| ADHD | N.S. | | | | | |
| OCD | N.S. | | | | | |

N_voxels_: number of voxels; Z: z-statistic; AAL: automated anatomical labelling; TD: typical developing; ASD: autism spectrum disorder; ADHD: attention deficit hyperactivity disorder; OCD: obsessive compulsive disorder; L: left hemisphere; R: right hemisphere; LING: lingual gyrus; CAL: calcarine gyrus; PHG: parahippocampal gyrus; FFG: fusiform gyrus; SOG: superior occipital gyrus; MOG: middle occipital gyrus; CUN: cuneus; IOG: inferior occipital gyrus; MTG: middle temporal gyrus

**References**

1 Lord C *et al.* The autism diagnostic observation schedule-generic: a standard measure of social and communication deficits associated with the spectrum of autism. *J Autism Dev Disord* 2000; **30**: 205–223.

2 Lord C, Rutter M, Le Couteur A. Autism Diagnostic Interview-Revised: A revised version of a diagnostic interview for caregivers of individuals with possible pervasive developmental disorders. *J Autism Dev Disord* 1994; **24**: 659–685.

3 Kaufman J *et al.* Schedule for Affective Disorders and Schizophrenia for School-Age Children-Present and Lifetime Version (K-SADS-PL): Initial Reliability and Validity Data. *J Am Acad Child Adolesc Psychiatry* 1997; **36**: 980–988.

4 Ickowicz A *et al.* The Parent Interview for Child Symptoms: A Situation-Specific Clinical Research Interview for Attention-Deficit Hyperactivity and Related Disorders. *Can J Psychiatry* 2006; **51**: 325–328.

5 Scahill L *et al.* Children’s Yale-Brown Obsessive Compulsive Scale: Reliability and Validity. *J Am Acad Child Adolesc Psychiatry* 1997; **36**: 844–852.

6 *Diagnostic and statistical manual of mental disorders (DSM-IV-TR)*. American Psychiatric Publishing, 2000.

7 *Diagnostic and statistical manual of mental disorders (DSM-5®)*. American Psychiatric Publishing, 2013.

8 Tzourio-Mazoyer N *et al.* Automated anatomical labeling of activations in SPM using a macroscopic anatomical parcellation of the MNI MRI single-subject brain. *Neuroimage* 2002; **15**: 273–289.
